# Cutaneous Redox Senescence

**DOI:** 10.3390/biomedicines12020348

**Published:** 2024-02-01

**Authors:** Mariáurea Matias Sarandy, Reggiani Vilela Gonçalves, Giuseppe Valacchi

**Affiliations:** 1 Department of Animal Science, Plants for Human Health Institute, North Carolina State University, North Carolina Research Campus, 600 Laureate Way, Kannapolis, NC 28081, USA; mmsouza@ncsu.edu; 2 Department of General Biology, Federal University of Viçosa, Viçosa 36570-900, MG, Brazil; reggiani.goncalves@ufv.br; 3 Department of Animal Biology, Federal University of Viçosa, Viçosa 36570-900, MG, Brazil; 4 Department of Environment and Prevention, University of Ferrara, 44121 Ferrara, Italy; 5 Department of Food and Nutrition, Kyung Hee University, Seoul 02447, Republic of Korea

**Keywords:** cutaneous senescence, reactive oxygen species, inflammation, biochemistry, molecular signaling

## Abstract

Our current understanding of skin cell senescence involves the role of environmental stressors (UV, O_3_, cigarette smoke, particulate matter, etc.), lifestyle (diet, exercise, etc.) as well as genetic factors (metabolic changes, hormonal, etc.). The common mechanism of action of these stressors is the disturbance of cellular redox balance characterized by increased free radicals and reactive oxygen species (ROS), and when these overload the intrinsic antioxidant defense system, it can lead to an oxidative stress cellular condition. The main redox mechanisms that activate cellular senescence in the skin involve (1) the oxidative damage of telomeres causing their shortening; (2) the oxidation of proteomes and DNA damage; (3) an a in lysosomal mass through the increased activity of resident enzymes such as senescence-associated β-galactosidase (SA-β-gal) as well as other proteins that are products of lysosomal activity; (4) and the increased expression of SASP, in particular pro-inflammatory cytokines transcriptionally regulated by NF-κB. However, the main targets of ROS on the skin are the proteome (oxi-proteome), followed by telomeres, nucleic acids (DNAs), lipids, proteins, and cytoplasmic organelles. As a result, cell cycle arrest pathways, lipid peroxidation, increased lysosomal content and dysfunctional mitochondria, and SASP synthesis occur. Furthermore, oxidative stress in skin cells increases the activity of p16^INK4A^ and p53 as inhibitors of Rb and CDks, which are important for maintaining the cell cycle. p53 also promotes the inactivation of mTOR-mediated autophagic and apoptotic pathways, leading to senescence. However, these markers alone cannot establish the state of cellular senescence, and multiple analyses are encouraged for confirmation. An updated and more comprehensive approach to investigating skin senescence should include further assays of ox-inflammatory molecular pathways that can consolidate the understanding of cutaneous redox senescence.

## 1. Introduction

As the aging process evolves, the body suffers from a reduction or loss in various functions and its ability to react to this process [1]. Although the intensity and progression of this process are related to intrinsic and extrinsic factors, its functional deterioration is directly linked to the development of different pathologies [2]. In this context, cardiovascular and respiratory disease [3,4,5], dementia [6,7], type 2 diabetes [8,9], bone and joint disorders [10,11], cancer [12], glaucoma [13], and skin diseases [14] are associated with the aging process.

Thus, understanding the main mechanisms and pathways activated during aging has been the subject of study by the scientific community for many centuries, whether through the search and discovery of the “fountain of youth” or through the search for a quality of life for an aging population and, consequently, a reduction in pathologies [15]. Previously, the mechanisms that promote aging were grouped into the following two classes: (1) genetic factors involved in development processes (cellular senescence, neuroendocrine responses, and immunological changes) and (2) the damage caused by the accumulation of epigenetic changes (somatic mutations associated with behaviors and environmental exposure). However, this separation is currently no longer acceptable since the two share similar characteristics found in different pathologies related to aging [16]. The term the epigenetic aging clock, for example, which describes DNA methylation patterns related to the measure of biological age [17], has been related to the epigenetic clock of senescence, and despite being considered separate processes, the similarity among them indicates that they are modulated by common activators [18]. Thus, canonical pathways of oxidative stress are common pathways of both aging and senescence and have been the target of interest among researchers in recent years [19]. Skin-related pathologies, for example, have been associated with ox-inflammatory processes and attributed to functional [19,20] or adaptations [21] with changes resulting from senescence during aging.

Therefore, given the importance of better understanding the connection between cutaneous processes, this review aims to bring together the state of the art of skin senescence redox regulation, identifying the main mechanisms involved in this process.

## 2. Aging and Senescence

Aging is a natural biological process marked by noticeable structural and morphological cellular changes and a gradual reduction in physiological functions over time [22]. Several theories have been proposed to explain the complex process of aging; however, there is currently a consensus that aging is directly associated with the negative regulation of metabolism, gene expression patterns, the exhaustion of the stem cell pool, dysfunction in intercellular communication, cellular senescence [23,24], and the production of high levels of reactive oxygen species (ROS) [25]. According to the latest United Nations Prospects (2022), life expectancy dropped in 2021 (71 years) compared to 2019 (72.8 years), with the impact of the COVID-19 pandemic being considered the leading cause. Still, the same report predicts that further reductions in mortality could result in global average longevity of around 77.2 years by 2050 [26]. One of the main causes for this optimistic outlook is related to advances in medical care and technologies that have allowed the development of innovative therapies, especially throughout the 20th century. Many variables can be considered to assess the increase in global life expectancy and the impact of aging on an individual’s health. However, skin aging has always been one of the most studied impacts, not only for aesthetic reasons but also because it is easily observed over time [2]. 

The clinical signs of skin aging include fine lines, dryness, sagging, coarse wrinkles, irregular pigmentation, and age spots [27]. The physiological and morphological changes that provide these clinical signs are mainly related to a reduction in cell proliferation and differentiation in the basal layer of the epidermis as well as in the dermis, resulting in thinner skin and, consequently, reduced barrier capacity [2]. Furthermore, with the reduction in these layers, cellular communication is also reduced, making it difficult for nutrients and oxygen to reach the superficial layers. These aspects, associated with the change in fibroblast functions as well as the synthesis of extracellular matrix (ECM) components, especially type I and III collagen and elastin, lead to the phenotype of wrinkles and loss of skin elasticity [28] and greater susceptibility to infections, dermatitis, wounds and several skin conditions [29].

Regarding the scientific scenario, understanding and identifying the process of aging and senescence and its particularities is essential in developing diagnostics and therapeutic strategies that can be applied to pathologies associated with aging. However, aging and senescence are independent processes where aging is time-dependent while senescence occurs throughout life, even before birth during embryogenesis [30]. Senescence was first identified in 1961 by Hayflick and Moorhead, who described it as an irreversible state of non-division where cells, despite no longer replicating (cell cycle arrest), remain metabolically active [31]. Since then, interest in the senescence process has increased, and evidence has been documented demonstrating that skin aging is significantly enhanced by the accumulation of senescent dermal fibroblasts [32]. Over the years, our cells multiply and renew as our body grows and through physiological processes of cell replacement, such as what occurs on the skin. In this process of cell division, telomeres, a terminal portion of the eukaryotic chromosome, are reduced in size. In dermal fibroblasts, for example, more than 30% of telomere length is lost, as well as a reduction in endogenous telomerase activity during adulthood [33,34].

## 3. Senescence Mechanisms

**Figure 1:**
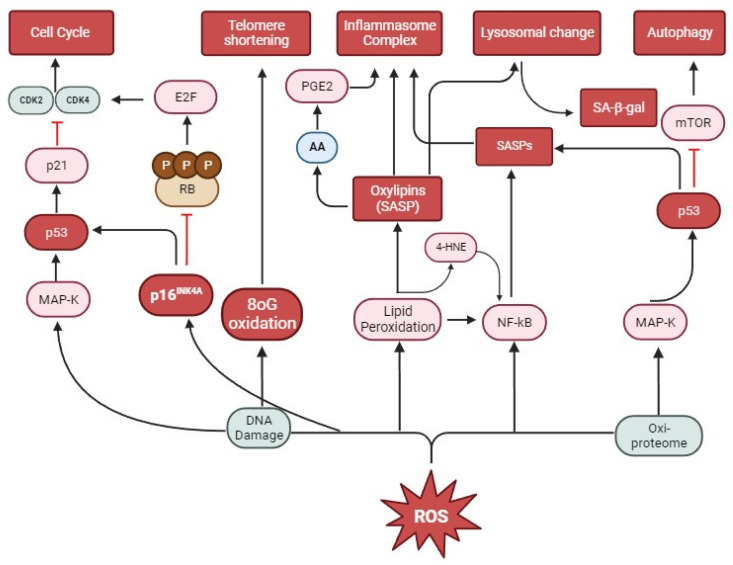
Main mechanisms involved in skin cellular senescence by oxidative stress.

The cellular senescence process can occur at different stages of development and in different physiological conditions, such as during embryogenesis [35] and wound healing [36]. It is worth mentioning that cellular senescence can have different origins, such as the shortening of telomeres (replicative senescence) which occurs over the years or can be prematurely induced by DNA damage, oxidative stress, oncogenic stress (especially the oncogenic activation of the MAP kinase pathway in fibroblasts which converts p53 into a senescence inducer), mitochondrial dysfunction (senescence associated with an increase in free radicals and ROS generation) [37], epigenetically (senescence induced by inhibitors of DNA methylases or histone deacetylases) [38], producing a phenotype senescence-associated secretory phenotype (SASP) (paracrine senescence) [39], and chemotherapy (therapy-induced senescence) [35] (Figure 1).

Although advances in knowledge about senescence reveal important benefits such as embryogenesis and tumor reduction due to cell cycle arrest, it is already established that senescent cells accumulate in tissues and organs during aging and are directly linked to the deficiency of physiological processes resulting from age and, consequently, significant pathologies related to aging. Relevant cellular changes, such as redox imbalance, the induction of cell cycle arrest, increased lysosomal density, the increased activity of the senescence-associated enzyme β-galactosidase (SA-β-gal), and secretion of cytokines and growth factors, known as phenotype senescence-associated secretion (SASP) are all hallmarks for cellular senescence [40]. The most widely used assay is the marker β-galactosidase activity at pH 6.0. Most normal cells express this enzyme at pH 4.0; therefore, to differentiate and mark only this enzyme in senescent conditions, pH 6.0 is used, which occurs in senescent cells [41]. SA-β-gal is a lysosomal hydrolase that catalyzes the hydrolysis of β-galactosides into monosaccharides such as 5-bromo-4-chloro-3-indolyl-b-D-galactopyranoside (XGal), which are then catalyzed to galactose and 5-bromo-4-chloro-3-hydroxyindole-1 [35]. This compound undergoes dimerization and forms a blue precipitate [42]. When the cell enters senescence, there is an increase in resident proteins such as β-galactosidase and increases in lysosomal activity, leading to an altered lysosome morphology [43]. Thus, this marker indicates SA-β-gal activity and suggests an increase in the lysosomal mass. Considering the false positive that this assay can sometimes give, it is always recommended to associate other tests with SA-β-gal activity.

Senescence-associated secretory phenotype (SASP) factors are secreted as the cells remain metabolically active; however, they are able to affect the neighboring cells and can compromise nearby tissues [44]. The secretion of these factors considerably alters the skin microenvironment, promoting age-associated chronic inflammation known as “inflammaging” [45,46]. Among the main SASP markers, there is also the transforming growth factor β (TGF-β), insulin-like growth factor 1 (IGF1), plasminogen activator inhibitor 1 (PAI1), and inflammatory cytokines and chemokines that can reinforce and propagate senescence in an autocrine and paracrine manner [35].

Furthermore, as cellular senescence is based on stable cell cycle arrest, the use of proliferation markers such as Ki67, the incorporation of 5-bromodeoxyuridine (BrdU), or typical nuclear changes such as heterochromatin have been reported as alternative approaches to evaluating cell senescence. The latter analysis is based on senescent cells’ mechanism to silence proliferation-promoting genes, organizing chromatin into structures called senescence-associated heterochromatin foci or SAHFs [41]. Along the same lines, the p16^INK4A^ protein is silenced in actively growing cells via histone H3 lysine 27 trimethylation (H3K27Me3) [47] and is highly expressed in senescent cells. However, these markers are considered complementary, as they alone are not sufficient to characterize the senescence process.

## 4. Skin Senescence

The skin is an extensive organ composed of the following two layers: the epidermis, which is external and highly cellular, and the dermis, which is internal, composed of cells, fibers, ground substance, blood vessels, and nerves [48]. The primary function of the skin is to provide a physical barrier against injuries from the environment. The epidermis, as an intensely cellularized layer, is divided into strata. The basal stratum is the deepest region, with active stem cells that transform into keratinocytes and melanocytes; the stratum spinosum is where we find cells with cytoplasmic projections similar to spines, and we also find dendritic cells; the stratum granulosum (or granular layer) has diamond-shaped epithelial cells; the stratum lucidum is present only in regions with thicker skin (for example, the palms of the hands and soles of the feet); and finally the stratum corneum is the outermost layer composed of keratin and dead keratinocytes (anucleated squamous cells) that release defensins, which are our first immunological defense [49]. The dermis, the innermost layer, is made up of a network of blood vessels, nerve endings, sebaceous glands, sweat glands, sensory nerves, and hair follicles. The main cell types present in this layer are fibroblasts, which synthesize and secrete important components of the extracellular matrix (ECM), providing structural integrity, resilience, and elasticity to the skin [50].

The different cell types found in the skin, when investigated individually, can be affected differently by the aging process, mainly because they are in different layers (superficial or deep) due to their diversity in metabolic and proliferative capacity [51]. Basal layer keratinocytes (stem cells), for example, express telomerase and retain their telomere length during UV-protected skin aging [52,53]. As the renewal process of this cell type occurs over 3 to 6 weeks, the elimination of cellular damage occurs quickly, thus preventing the accumulation of damage and making the tissue compartment comparatively resistant to stress [54]. Furthermore, keratinocytes have the ability, during exposure to high levels of oxidative stress, to activate apoptotic mechanisms as a response to DNA damage [55]. However, different cellular senescence markers such as SA-β-Gal [56], p16^INK4A^ [57], and p21 [52] were found in keratinocytes from aged donors, suggesting that despite the characteristics of cellular renewal predominant in this cell type, there is a close relationship between aging and senescence. On the other hand, melanocytes located in the basal stratum of the epidermis have high levels of p16^INK4A^ to combat ROS formed as a byproduct during melanin production, regardless of age or cell cycle regulation [58,59]. In addition, the fibroblasts, which are predominant cells in the dermis, are cells that proliferate slowly compared to epidermal cells and, therefore, may be more prone to the accumulation of DNA damage and, consequently, senescence [60]. Also, reduced protein metabolism in aged fibroblasts can lead to the accumulation of misfolded proteins [61], leading to the senescence of this cell type [62]. 

However, it has already been observed that these cells form an intercommunicative network that directly interferes with the homeostasis of this cellular network, contributing to the general decline of the tissue [63]. In cutaneous tissue, aging is characterized by photodamage and a reduction in the thickness of the epidermis, mainly impairing its physical barrier function [64], as well as a reduction in the cellular and fibrillar components of the dermis, compromising resistance and tissue elasticity [65]. Due to their capacity for constant cell renewal, the permanence of senescent cells in the skin can be considered low [66]; however, the renewal of epithelial cells is reduced in the elderly, which is why senescent cells can accumulate over time [67]. It is already known that aged skin can contain up to 50% of senescent cells in the dermis [68,69]. In this context, despite the existence of different processes, the chronic accumulation of senescent cells in the skin (and other tissues) contributes to characteristic aging phenotypes, as well as tissue dysfunction [30], which can make it difficult to differentiate between skin layers in the aging and senescence process.

Senescence in the cells of the basal layer of the skin can be activated mainly by exhaustive cell cycles, leading to telomere shortening, as well as DNA damage response [70]. As a consequence, senescence reduces signaling for basal cell proliferation and, consequently, reduces the differentiation of keratinocytes, altering the thickness of the epidermal layer and compromising its functionality, especially that of the physical barrier [71]. Furthermore, cytokines from SASP, such as IL-6, are stimulated after continuous damage to the DNA of keratinocytes and melanocytes [72], and these cytokines are also indicators of aging [73].

In addition to changing the thickness of the epidermis as well as reducing the physical barrier function, another important skin aspect related to senescence is skin pigmentation [74]. Melanin is formed due to pigments produced by melanocytes, which are capable of absorbing UV light and protecting the skin from UV radiation [75]. However, as senescence promotes cell cycle arrest and, consequently, reduces proliferation, the number of melanocytes decreases, and skin color also lightens in areas protected from the sun [52], resulting in spots and reduced barrier function to radiation. On the other hand, melanin accumulation accelerates melanocyte senescence via p16^INK4A^ [76]. Proteins such as p16^INK4a^, p21CIP1, and p53 accumulate over the years in skin cells [52] in response to cellular damage and can regulate the cell cycle; therefore, they are important indicators of senescence in the skin and other tissues [77].

In the dermis, senescent fibroblasts (p16^INK4A^-positive) reduce the synthesis and release of IGF-1 [32], suppressing collagen synthesis and promoting epidermal atrophy. Keratinocytes have receptors for IGF-1, which stimulate the proliferation and differentiation of this cell type, demonstrating how changes in individual cells can interfere with dermal–epidermal dynamics and the general decline in skin tissue [78]. Furthermore, premature senescence was found in the fibroblasts of vitiligo skin, which, despite experiencing a greater production of fibronectin, does not exert the usual barrier function, resulting in impaired cell adhesion [79]. In this same study, it was observed that fibroblasts secrete and release growth factors associated with skin aging, such as the hepatocyte growth factor (HGF) and inflammatory cytokines such as interleukin 1-beta (IL-1b), which controls the functionality of melanocytes, negatively regulating the expression of E-cadherin and impairing the integrity and architecture of epithelial tissue [80]. 

As they are metabolically active cells, senescent fibroblasts [81] and keratinocytes [32] secrete greater amounts of metalloproteinases (MMPs) that degrade the dermal matrix and are also detected in chronologically aged and photoaged skins [82]. Furthermore, with aging, fibroblasts reduce their proliferation capacity (cell cycle arrest) and release cytokines that are part of SASP, increasing inflammatory processes as well as driving skin aging [32]. Other pathways that can activate senescence in dermal fibroblasts derive from telomere shortening (after several replication cycles), mitochondrial dysfunction (in response to the external stressors triggering oxidative stress) [37], and responses to DNA damage that leads to permanent or transient cell cycle arrest [32].

## 5. Contributors (Extrinsic and Intrinsic Factors) 

It is well-established that chronic exposure to stressors can induce senescence in skin cells [65,83]. This phenomenon can be affected by both intrinsic and extrinsic factors. Intrinsic factors such as genetic predisposition to metabolic alteration or deficiency in the synthesis and degradation of a specific amino acid/protein [84] or hormones [2] and skin pH [85] are genetically inherited factors. Extrinsic factors (environmental influences, nutrition, and lifestyle) have been shown to play a key role in skin senescence by promoting telomere shortening, mitochondrial impairment as well as DNA damage [65]. Exposure to environmental pollutants such as the ozone (O_3_) [86], particulate matter (PM) [87], sunlight (UVB and UVA) [88], diesel engine exhaust (DEE), as well as some lifestyle factors (diet, smoking, and physical activity) [65] have direct implications towards skin health and senescence [89]. However, it can sometimes be difficult to separate intrinsic and extrinsic causes when it comes to skin cell aging and senescence, as it is impossible to rule out the environmental components that cause skin senescence.

UV-induced senescence has been associated with increased reactive oxygen species (ROS) production. In both in vitro and in vivo models, exposure to UVB causes DNA damage, consequent cell cycle arrest, increased senescence-associated β-galactosidase (SA-β-Gal), p16^INK4A^ activity, and p21Waf-1, p53 activation in fibroblasts and keratinocytes [90,91], which activate SASP and increase the skin’s inflammatory potential [92]. The above-mentioned literature shows that senescent cells occur mainly in the epidermis but not in the dermis, demonstrating that external factors promote senescence. Endogenous damage accumulated over the years due to ROS production may have a genetic origin. Still, it can also be caused by an unfavorable lifestyle, including an unhealthy diet [92,93], which can trigger dermal cellular senescence. These data allow us to infer that the origin of the stressor (extrinsic or intrinsic) can have different skin cell-type targets. UVA exposure can lead to the generation of ROS such as singlet oxygen (^1^O_2_), hydroxyl radical (OH^.^), superoxide anion (O_2_^−^), and hydrogen peroxide (H_2_O_2_) via their interaction with specific chromophores [94]. UVA is especially active in the presence of oxygen and endogenous photosensitizers (porphyrins, heme-containing proteins). The photoexcited sensitizer can react with molecular oxygen, leading to the formation of both singlet oxygen (^1^O_2_) and superoxide radical anion (O_2_^−^). Superoxide radical anion (O_2_^−^) can incur enzymatic dismutation and form H_2_O_2_, directly interacting with biomolecules, including DNA bases. In addition, it has been suggested that (O_2_^−^) can induce the release of free iron and heme iron in cells and further implement oxidative stress levels via the Fenton reaction. This reaction can then lead to a cascade of effects that can further promote the expression of pro-inflammatory cytokines and mutations in mitochondrial DNA and activate the transcription of genes encoding matrix metalloproteinases [95,96]. In this context, mutations in mitochondrial DNA and increased ROS have been associated with the progression of photoaging and skin senescence [96,97]. 

Ozone (O_3_) is a secondary pollutant, and although it does not penetrate the skin, it induces oxidative stress in the cells of the stratum corneum of the epidermis, producing H_2_O_2_, lipid peroxidation, and protein oxidation [98]. Typically, these harmful effects are associated with decreased levels of antioxidants in the surface layers, such as vitamin E and vitamin C [99]. After exposure to the ozone, the product of lipid peroxidation in the stratum corneum activates phospholipases that release arachidonic acid [100,101], which is converted into several types of prostaglandins, especially PGE-2, a potent inflammatory mediator that promotes cellular senescence [102]. However, despite studies demonstrating an increase in PGE in senescent cells [103,104], there is still a gap in our knowledge about the mechanisms by which PGEs activate senescence in keratinocytes. An indication of a possible pathway for senescence activation via PGEs is due to the fact that they are considered part of the SASP, which is an important marker and initiator of cellular senescence and inflammatory processes [101], suggesting this pathway is the main senescence activation mechanism. Furthermore, studies with fibroblasts indicate the role of PGE-2 in inducing senescence [102,105]. Recent evidence has shown that prolonged exposure to O_3_ is associated with premature skin aging [83]. Studies carried out by our group demonstrate that exposure to O_3_ leads to the release of lactate dehydrogenase, reduced cell proliferation, lipid peroxidation, and the activation of NF-κB [99,106], in addition to amplifying markers of oxy-inflammation in explants UV-induced skin damage [107]. Interestingly, a retrospective study collecting human patient data reported an increase in emergency room visits for conditions such as urticaria, eczema, and contact dermatitis at an increased level of ambient ozone exposure [108], and the harmful cutaneous effect of the ozone was also recently demonstrated in a clinical study [109].

Particulate matter (PM), another environmental stressor, is composed of highly lipophilic particles that, in some circumstances, might be able to penetrate the skin and induce senescence in keratinocytes by reducing the methylation of the canonized senescence marker p16^INK4A^ as a specific inhibitor of CDK4/CDK6 [110]. The DNA damage response (DDR) and ROS formation are responsible for the decrease in the expression of DNA methyltransferase (H3K27Me3), a transcriptional suppressor, leading to the hypomethylation of the p16^INK4A^ promoter region. This is one of the main mechanisms used by ROS for the induction and maintenance of the cellular senescence process in skin cells. Similarly, cigarette smoke induces cellular senescence (SA-β-gal-positive) in the skin via pathways mediated by oxidative stress [111]. In response to the oxidative damage caused by cigarette smoke, epithelial cells can activate nuclear factor-kB (NF-kB) [111], a crucial transcription factor in the induction of SASP, increasing the levels of pro-inflammatory cytokines during senescence. Furthermore, nicotine increases the proportion of α3 nAChR channels in fibroblast membranes, altering Ca^2+^ metabolism in these cells, leading to reciprocal changes in cell cycle progression and function [112] and culminating in the process of senescence.

Other stressors to be considered are those of endogenous origin, such as metabolic changes [113], inflammatory diseases, aging [114], hormonal imbalance [115], and changes in the cellular redox environment [30]. These stressors, hereditary or not, commonly induce senescence due to an inadequate mitochondrial function, the increased generation of ROS, DNA damage triggering, or the activation of tumor suppressor genes [116]. Furthermore, the high capacity of skin cells for proliferation and/or increased ROS followed by inflammation promotes cumulative damage that leads to telomere shortening [117].

Thus, considering that under normal conditions, we are not exposed exclusively to a single stress factor but simultaneously to a combination of different ones, both intrinsic and extrinsic, associated or not, there is a consensus that they all share a common part in the activation of skin cellular senescence and in the increase in the generation of reactive oxygen species (ROS) [118].

## 6. Oxidative Stress and Senescence 

Due to the skin’s ability to regenerate, much of the damage caused by environmental stressors is transient, but continuous exposure associated with genetic factors and time activates the production of reactive oxygen species [119]. Since the skin is an external organ that requires constant cell renewal, the skin comprises different layers of metabolically active cells with high energy consumption [120]. In this context, mitochondria play a fundamental role in producing energy in the form of ATP for cells. However, this process results in increased ROS production [118,121,122]. 

These reactive molecules act on cells in the skin in response to different intrinsic and extrinsic stressors, activating the natural endogenous antioxidant defense [123]. Antioxidants are present in different layers of the skin. In the more external layer (epidermis), we find higher concentrations of antioxidants than in the innermost layer (dermis); however, in the stratum corneum of the epidermis, vitamins C and E (αγ and α-tocopherol), GSH and uric acid can be found [124]. However, with continuous stimuli, an imbalance occurs, and ROS production exceeds the synthesis of antioxidant enzymes, which becomes insufficient to neutralize the harmful effects of ROS. At this stage, tissue oxidative stress begins. The consumption or topical application of exogenous antioxidants helps to balance this effect [125,126,127]. Yet, the increase in ROS can activate different pathways via reacting with various compounds and participating in several redox reactions that drive cellular senescence in the skin. As a highly reactive species, ROS destroys cellular macromolecules such as proteins, lipids, and nucleic acids. Among the mechanisms modulated by ROS that induce senescence, the one that was most studied and, therefore, most consolidated is “DNA Damage” [128], probably because one of the main characteristics of senescence is the arrest of the cell cycle due to the shortening of telomeres or due to some damage to the DNA [128]. However, recent studies have identified that, before causing damage to the DNA, ROS targets the proteome first. During the oxidation of these proteins, the “oxi-proteome” prevents the repair of damaged DNA [129,130]. Around 35% of the proteins accumulated in senescent fibroblasts depend on oxidative processes [87], and this phenomenon has been associated with aging disorders [131]. The SIRT1 protein, for example, is an NAD^+^-dependent enzyme known as an autophagy initiator, and when suppressed by ROS, it induces senescence in keratinocytes [132]. 

Telomere shortening, a hallmark of senescence, also occurs through oxidative damage [133]. ROS can either directly damage mitochondrial DNA (mtDNA) or cause damage when in synergy with telomere damage [131]. This damage occurs due to a large amount of guanine present in telomeric DNA (8-hydroxyguanine) associated with the great affinity of ROS to oxidize guanine (8-oxoguanine (8oG)). Furthermore, ROS inhibits telomerase activity [134] and induces single-strand breaks (SSBs) on telomeres [133] (Figure 1). However, cellular senescence can be a great ally with anti-tumorigenic effects when it interrupts the cell cycle and, consequently, the proliferation of damaged or dysfunctional cells. Studies using murine models have highlighted the high prevalence of senescent cells in pre-malignant tumors, thus explaining the slow growth and low malignancy of these tumors [135]. In this model, senescence was associated with the activation of tumor suppressor proteins, such as p53, p16^INK4A^, and ARF, and their loss or inactivation led to tumor-malignant progression [136,137,138]. Furthermore, system cells rapidly eliminate senescent tumor cells, resulting in efficient tumor regression [135,139].

When cells activate DNA repair mechanisms, permanent structural and functional changes, such as senescence, trigger proteins with multiple functions, for example, the retinoblastoma (RB) family and the p53 proteins. Thus, the activation of tumor suppressors such as the RB protein and the p53 transcription factor via continuous ROS stimulation stimulates oncogene-induced senescence. In normal pathways, CDKs phosphorylate retinoblastoma proteins (RB1, RBL1, RBL2), reducing their ability to inactivate E2F, which is a protein necessary for cell cycle progression. However, during the senescence process, a CDK inhibitor known as p16^INK4A^ prevents RB phosphorylation, repressing E2F and preventing cell cycle progression [140]. Furthermore, p16^INK4A^ activates p53, which inhibits autophagy-inducing mTOR kinase (the mammalian target of rapamycin) [141]. However, although p16^INK4A^ is a widely used marker for senescence, it may not be expressed by all senescent cells [104], thus revealing the importance of using multiple markers to detect senescence. p53, a protein encoded by the TP53 gene, also inhibits CDK4 and CDK2 kinases via p21, inducing cell cycle arrest [105]. When subjected to prolonged stress, excessive ROS production activates p53, suppressing the process of autophagy followed by apoptosis mediated by mTORC1, resulting in the accumulation of damaged cells [139] and the induction of the senescence process. A study carried out with murine models observed that, in adipose tissue, skeletal muscle, and eyes treated with a compound that removes senescent cells positive for p16^INK4A^, there was a delay in the onset of pathological phenotypes associated with aging. These findings indicate that cellular senescence is directly linked to age-related phenotypes and that the removal of senescent cells can prevent or delay aging-related pathologies and prolong life expectancy [15].

However, the many changes associated with the senescence process in the skin are directly related to mitochondria. The phosphorylation of the ATM, AKT, and mTOR complex increases mitochondrial biogenesis and, consequently, the DNA damage response (DDR) mediated by ROS. This promotes cell cycle arrest and SASP development and demonstrates the role of this organelle in both ox-inflammatory and DDR processes. SASPs, mainly characterized by pro-inflammatory cytokines, are transcriptionally regulated by NF-κB and CEBP-β in response to ROS-induced DNA damage, and their expression is maintained by autocrine feedback [141]. Thus, the activity of autophagy lysosomes and mitochondria are functionally interconnected during senescence. Under normal conditions, dysfunctional mitochondria undergo autophagy through lysosomal compartments [107]. However, the increase in ROS activates ataxia-telangiectasia mutation (ATM), a predominantly nuclear protein that is a member of the serine/threonine kinase family of proteins. This activated protein modulates p53-mediated DNA damage response targets [142] but also increases lysosomal acidity mediated by signaling extracellular signal-regulated kinase (Erk) activity, compromising the normal autophagy flow [143]. Thus, mitochondrial autophagy is hampered, and consequently, there is an accumulation of dysfunctional mitochondria and excessive production of ROS. The increase in lipid peroxidation during oxidative stress increases the content of intra-lysosomal lipofuscins in fibroblasts during senescence [19]. Furthermore, the increase in lysosomal-resident proteins such as β-galactosidase, associated with the delivery of autophagic proteins via the macro-autophagy (MA) and chaperone-mediated autophagy (CMA) pathways that occur in response to increased ROS [111], is also responsible for the change in lysosomal mass. It is noteworthy that the lysosomal β-galactosidase activity identified at pH 6.0 (SA-β-gal) is a byproduct generated via senescence and not by what causes cellular senescence [43]. 

ROS can also modulate skin senescence by activating SASPs. This complex mixture of factors can be produced as an ROS-stimulated DNA damage response [144]. Senescent cells can maintain the synthesis of SASP markers such as IL-6, TGF-β, VEGF, CCL2, and CCL20, which can reinforce senescence in an autocrine and paracrine manner [39]. Furthermore, it is reported that SASP factors that promote paracrine senescence, such as IL-1α and IL-1β, activate the inflammasome since IL-1β is only formed after activation via the inflammasome multiprotein complex. This complex is formed by caspase 1 and adapter molecules, such as NRLs and ASC [145,146,147]. Therefore, the synthesis of SASP factors is indisputably related to inflammatory diseases caused by senescent cells during the senescence and skin aging process. Within SASP, the 4-hydroxy-2-nonenal (4-HNE) is another molecule associated with inflammatory processes. 4-HNE is a highly reactive product of lipid peroxidation caused by the action of ROS, which can form adducts with cellular proteins and even DNA [148]. Human keratinocytes exposed to O_3_ inhibited cell proliferation and stimulated increased oxidative damage, which increased levels of 4-HNE and activated the NF-κB pathway, demonstrating that O_3_ is a potential inducer of skin inflammation [99]. The continuous action of this stressor has been associated with premature aging and senescence [149] (Figure 1).

Although the standard and alternative mechanisms that trigger senescence are well established, some gaps in the research still exist, and, as there is no single reliable marker for the detection of senescence in vivo, it is the consensus of the International Cell Senescence Association (ICSA) that at least one combination of more than two markers is used to identify a cell as senescent [40].

## 7. Oxylipins and Senescence

Lipids found in the skin are related to functions such as the epidermal lipid barrier, which prevents water loss and the entry of harmful substances [150], and also cell signaling, which ranges from membrane trafficking to the transduction signal [151]. This is in addition to the role of skin hydration through lipids produced in the sebaceous glands [152]. Therefore, some oxylipins are important for maintaining the biological functions of the skin, and knowing the molecules and pathways that activate the formation of these bioactive lipids can direct effective therapies. In this context, the role of Lipoxygenases (LOX), which are key enzymes in the synthesis of different oxylipins in various biological processes, has been investigated [153]. In the skin, for some decades now, LOX has been associated with the modulation and maintenance of the permeability barrier [154]. LOX oxidizes esterified structural lipids, such as ceramides, present in the corneal layer, and its deficiency negatively affects lipid levels essential for the barrier, as well as cornified envelope proteins [155].

However, in recent decades, the chemical or enzymatic modifications of lipids called epilipidoma [156] have been considered fundamental in understanding skin changes, including both pathological and common physiological changes in age. Furthermore, as already mentioned, 4-HNE is a widely used marker to characterize increased lipid peroxidation and the resulting accumulation of lipid-derived aldehydes in senescent cells [157] (Figure 1). In this session, we highlight the involvement of lipids resulting from oxidative stress, oxylipins [105], and their role in the senescence of skin cells.

Lipofuscin (LF) is a type of oxylipin found in lysosomes in pigments called the “age pigment” because they are frequently found during aging [158], promoting the senescence of skin cells [30]. LFs accumulate in lysosomes, leading to an increase in the lysosomal lumen. The presence of the already consolidated senescence marker SA-β-Gal and an increase in lysosomal mass have been associated with the presence of lipofuscin in senescent epithelial cells [159]. In this context, the interruption of the normal metabolic function of lysosomes, as well as the deficiency of the autophagy process, contribute to the maintenance of this cellular state [160].

Different oxylipins were identified in the composition of SASP, called SASP lipids [161]. These oxylipins, in addition to the characteristic pro-inflammatory role of SASPs, prevent senescent fibroblasts from being eliminated by the innate immune system, reducing the signaling mediated by Toll-like receptor (TLR) 2 and TLR6 in macrophages [162]. Arachidonic acid (AA), as well as prostaglandins (PGD2 and other variants), and leukotrienes are well-known lipid SASPs. Cyclooxygenase 2 (COX2) converts AA to PGD2, which activates a signaling cascade involving p53, which is a marker of cellular senescence [105,163]. Like prostaglandins, leukotrienes are synthesized from arachidonic acid cleaved during the oxidation of membrane phospholipids [164] and increase during cellular senescence [165]. Recent data demonstrate that increased lipid peroxidation drives increased metabolism, import, and lipid accumulation in senescent cells (SA-β-Gal-positive). Thus, oxidative imbalance promotes lipid peroxidation in skin cells, initiating a positive feedback cycle that maintains or triggers senescence, regardless of the persistence of other stressors [166].

## 8. Strategies to Prevent Skin Senescence

The search for understanding the different triggering factors and mechanisms involved in skin senescence has driven the investigation of different strategies to eliminate or prevent this cellular condition. These approaches are known as “senotherapy” or “senotherapeutic strategies” [167] and can be based on the following two distinct pathways: (1) senolytic pathway: the selective induction of cell death, used especially in cancer treatment (chemotherapy) [168] and (2) senomorphs, to control deleterious effects through SASP inhibition. However, a third strategy, “immune system enhancement”, has been proposed to minimize the damage of skin senescence [169]. It is worth mentioning that there are currently no officially clinically approved medications considered a “gold standard” in the treatment of skin senescence [30].

The clear relationship between ROS levels and senescence has sparked great interest in research into antioxidant compounds. Senotherapy based on antioxidants of natural or synthetic origin has shown promising results [170]. Synthetic flavonoids such as apigenin and kaempferol [171], as well as glucocorticoids and cortisone [172], regulate senescence through senomorphic mechanisms, inhibiting the activity of NF-κB-p65, which are activators of SASPs in skin cells. Ramrammycin, an mTOR inhibitor, also reduces the synthesis of SASP (IL-6) by suppressing the NF-κB pathway [173].

Resveratrol was investigated due to its potent antioxidant and anti-inflammatory capacity in senescent skin cells and induced a reduction in SASP by increasing the expression of AMPK-FOXO3 [174]. However, some studies have demonstrated that resveratrol also induces apoptosis and cellular senescence in primary and cancer cells through the activation of DNA damage response [175,176]. Other synthetic compounds that reduce SASPs are UR-13756 and BIRB 796 [177], which act by inhibiting the p38 MAPK pathway. Thus, the main signaling pathways that modulate SASP appear to converge on the NF-κB pathway, demonstrating the importance of this pathway for the study of senescence.

The investigation of synthetic compounds, such as ferulic acid [178] and quercetin [179], and natural compounds, such as nectrandrin B [180], used as senotherapy, demonstrates action that aims to maintain and progress the cell cycle, reversing senescence through a reduction in ROS and inhibition of the p16, p21, p27, p53 pathways. Nectrandrin B [180] is a natural compound composed of six herbs (Rehmannia glutinosa, Panax Ginseng, Asparagus cochinchinensis, Ophiopogon japonicus, Cortex Lycii., Poria cocos) [181] and Yang Yan Qing E Wan (YYQEW) [182].

In relation to the already-known effect of oxylipins on lysosomal compartments, induced autophagy was identified as an important therapeutic target in preventing cellular senescence [183]. In this context, caffeine was reported as a potent activator of signaling pathways for autophagy and the reduction in ROS levels; however, it may also benefit tumor cell growth [184]. The strategies presented in this session can be seen in Table 1.

In addition to the different strategies mentioned above, it is important to highlight that the skin’s antioxidant system is composed of enzymatic substances (glutathione peroxidase (GPx), catalase (CAT), and superoxide dismutase (SOD)) and non-enzymatic substances (such as estradiol and melatonin, vitamins E and C) [185]. In addition to the known action of SOD in neutralizing reactive molecules, there are reports that vitamin C positively modulates the expression of SOD and prevents collagen degradation [186]. It is already known that vitamins that act as skin antioxidants are mainly taken from food [93,187]. Furthermore, foods such as Ginseng [188], the seed of *Prunus mume* [189], in addition to the consumption of probiotics and prebiotics [190], have demonstrated efficiency in modulating the endogenous antioxidant capacity of the skin, reducing oxidative damage and, consequently, aging and/or senescence. These studies indicate that, in addition to the topical use of synthetic or natural formulas, a complementary strategy should be promoted for the consumption of functional foods to combat senescence and aging [191]. It is worth mentioning here that the consumption of synthetic antioxidants in high doses can harm the body and activate oxidative stress, and the safest way to ingest these antioxidants would be in the form of an adequate diet [191,192].

Thus, identifying new mechanisms as a therapeutic target, as well as identifying new bioactives that prevent telomere loss, cell cycle arrest, and increased ROS alongside inflammasome activation, is a new and exciting area of investigation that may well offer new treatments against skin senescence.

## 9. Conclusions

The redox imbalance promoted by the increase in ROS resulting from continuous stressful stimuli overloads the endogenous antioxidant enzymatic system, boosting the senescence process in the skin. ROS activate different pathways that culminate mainly in oxidative damage to telomeres and also in damage to DNA, activating pathways that block CDks, especially p16^INK4A^ (retinoblastoma protein inhibitor) and p53, preventing cell cycle progression. Furthermore, lipid peroxidation, as a result of ROS, alters the content and, consequently, the lysosomal morphology of SA-β-gal. These events compromise the processes of autophagy and apoptosis by silencing proteins, such as mTOR and increasing the number of dysfunctional organelles in the cytoplasm such as mitochondria. The mechanistic basis of cutaneous inflammatory processes in senescent cells is directly related to the production of SASP and the formation of oxylipins, which, in a positive feedback process, activates the NF-κB pathway, promoting the formation of the inflammasome complex, releasing different pro-inflammatory factors and cytokines. However, these markers, in general, must be evaluated together, as they are not reliable when investigated in isolation.

Therapeutic strategies called senotherapy have investigated the action of different compounds. Yet, more studies are needed to verify their short and long-term effects, understand the molecular mechanisms that involve redox action in senescent cells, and provide new biomarkers that mainly identify therapeutic targets to combat-related pathologies.

## Figures and Tables

**Table 1:** Main strategies and therapeutic targets for skin cell senescence.

References	Skin Cell	Main Senescence Markers	Senotherapeutic Strategy	Intervention Origin	Main Senotherapeutic Target	Mechanism of Action
[171]	Human fibroblast	BrdU and SA β-gal	Flavonoids (apigenin and kaempferol)	Synthetic	Reduced SASP inhibited NF-κB p65 activity	Reduced inflammation
[172]	Human fibroblast	BrdU and SA β-gal	Glucocorticoids cortisol and corticosterone	Synthetic	Reduced SASP inhibited NF-κB p65 activity and suppressed IL1A	Inhibited the ability of SASP to stimulate tumor cell invasion
[173]	Human fibroblast	SASP components	Rapamycin	Synthetic	Reduced SASP inhibited NF-κB	Inhibited cell proliferative activity
[174]	Human Keratinocytes	SA β-gal	Resveratrol	Synthetic	Reduced SASP increased Activation of AMPK-FOXO3	Inhibited cell proliferative activity
[184]	Human Keratinocytes	ROS	Caffeine	Synthetic	A2AR/SIRT3/AMPK-mediated autophagy and ROS reduction	Reduced ROS increased proliferative activity
[177]	Human fibroblast	BrdU, SA β-gal and SASP components	UR-13756 and BIRB 796	Synthetic	Reduced SASP inhibited p38 MAPK and MAPKAPK2	Reduced inflammation
[178]	Human fibroblast	ROS and SA β-gal	Ferulic acid	Synthetic	Increased proliferation and cell cycle progression	Increased proliferative activity and antioxidant effects
[180]	Human diploid fibroblasts	ROS and SA β-gal	Nectandrin B	Natural	Cell cycle progression, reduction in p16, p21, p27, p53	Increased proliferative activity and antioxidant effects
[181]	Human fibroblast	SAHF and SA β-gal	Rehmannia glutinosa, Panax Ginseng, Asparagus cochinchinensis, Ophiopogon japonicus, Cortex Lycii., Poria cocos	Natural	Cell cycle progression, reduction in p16, p21	Improved skin morphological parameters
[182]	Human fibroblast	ROS, SA β-gal and p16^INK4a^	Yang Yan Qing E Wan (YYQEW)	Natural	Cell cycle progression, reduction in p16 ^INK4a^, and reduced ROS	Increased antioxidant effects
[179]	Human fibroblast	ROS and SA β-gal	Quercetin	Synthetic	Cell cycle progression, reduction in p16, p53, and reduced ROS	Increased antioxidant effects

SA β-gal: senescence β-galactosidase; SAHF: senescence-associated heterochromatin foci; ROS: reactive oxygen species.
